# Functional Characterization of Squalene Epoxidases from *Siraitia grosvenorii*

**DOI:** 10.3390/plants14121740

**Published:** 2025-06-06

**Authors:** Huan Zhao, Ze Song, Xuan Liu, Shukun Gong, Qi Tang, Changli Liu, Yifeng Zhang, Xianan Zhang, Haiyun Gao, Wei Gao, Yating Hu, Luqi Huang

**Affiliations:** 1 School of Traditional Chinese Medicine, Capital Medical University, Beijing 100069, China; huanzhao@ccmu.edu.cn (H.Z.); gxszsnld@163.com (Z.S.);; 2 College of Pharmacy, Chengdu University of Traditional Chinese Medicine, Chengdu 611137, China; 3 College of Horticulture, Hunan Agricultural University, Changsha 410128, China; 4 State Key Laboratory for Quality Ensurance and Sustainable Use of Dao-di Herbs, National Resource Center for Chinese Materia Medica, China Academy of Chinese Medical Sciences, Beijing 100700, China; 5 Key Laboratory of Sustainable Utilization of Traditional Chinese Medicine Resources in Jiangxi Province, Institute of Traditional Chinese Medicine Health Industry, China Academy of Chinese Medical Sciences, Nanchang 330115, China

**Keywords:** *Siraitia grosvenorii*, *ERG1* knockout, mogroside, squalene epoxidase

## Abstract

The medicinal plant *Siraitia grosvenorii* produces sweet-tasting cucurbitane-type mogrosides from the atypical triterpenoid precursor 2,3,22,23-dioxidosqualene (SDO), rather than the conventional 2,3-oxidosqualene (SQO). However, SDO formation in mogroside biosynthesis remains unclear. Here, we systematically characterized two squalene epoxidases (SgSQE1/2) through phylogenetic analysis, heterologous expression, subcellular localization, qRT-PCR, and alanine scanning studies. Both *Sg*SQE1 and *Sg*SQE2 exhibited squalene epoxidase activity, with *Sg*SQE2 catalyzing SDO formation in yeast. We identified two critical catalytic residues governing epoxidation efficiency through mutagenesis. Both *Sg*SQEs were localized in the ER, while expression profiling revealed a similar trend between *SgSQE2* expression and mogroside accumulation in fruits. In our study, we developed a genomically engineered strategy for heterologous SQE characterization. These results lay the foundation for the SQE catalytic reaction involved in mogroside biosynthesis, and provide gene resources and a feasible approach for triterpene metabolic engineering.

## 1. Introduction

*Siraitia grosvenorii* (Swingle) C. Jeffrey ex A. M. Lu & Zhi Y. Zhang, a unique medicinal and sweetening plant native to China, belongs to the Cucurbitaceae family. Mogrosides are the major bioactive and sweetening components isolated from the fruit of *S. grosvenorii*, and they have significant potential in the food and medicine fields. Mogrosides possess notable pharmaceutical activities such as anti-inflammatory effects [1,2,3], anticancer potentials [4], hepatoprotective properties [5,6], and hypolipidemic activity [7]. Mogroside V could reduce astrocyte inflammation and exhibit neuroprotective effects after cerebral I/R injury [2], and attenuate chronic toxicity induced by nanoplastics [8]. Mogroside ⅡE could improve pancreatitis in cell and mouse models [9]. Mogroside V showed β2-adrenergic receptor-targeted bronchodilator activities, suggesting potential drugability for receptor-mediated respiratory ailments like asthma [10]. Mogroside V and its aglycone mogrol show significant neuroprotective effects and metabolic regulation capabilities in Parkinson’s mouse models. Furthermore, mogrosides possess anti-diabetic properties [11,12], particularly in improving gut microbiota dysbiosis in type 2 diabetic (T2DM) rats [11]. Meanwhile, as naturally sweet compounds, mogrosides reached a 300-fold sucrose-relative sweetness [13]. With the advantage of high sweetness and low calories, mogrosides have been globally approved as safe food additives in Japan (1995), China (2007), U.S. (2009), and the European Union (2010) [14], enabling widespread use as zero-calorie sugar substitutes to reduce sucrose content in food products. Researchers transformed mogrosides biosynthetic genes into vegetables making the transgenic cucumber and tomato produce mogrosides, which could improve the flavor of vegetables [15].

**Figure 1:**
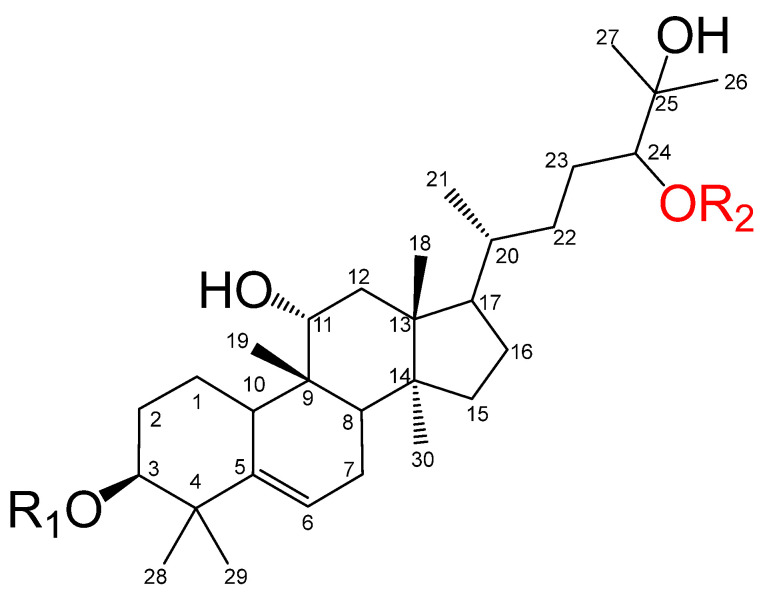
Chemical structure of cucurbitane-type triterpene from *S. grosvenorii*. Mogrol: R1/R2 represent H; mogrosides: R1/R2 represents different number of glucose.

Mogrosides represent a typical cucurbitane-type tetracyclic triterpenoid with the common aglycone, mogrol, which features four regionally specific hydroxyl groups at C3, C11, C24, and C25. Among them, C3-OH and C11-OH have been widely observed in most triterpenes, C3-OH was generated from the epoxy group during the cyclization of 2,3-epoxysqualene, while C11-OH was introduced by relatively conserved CYP450s, such as CYP87D18 from *S. grosvenorii* [16], CYP87D20 from *Cucumis sativus* L. [17], and CYP88D6 from *Glycyrrhiza uralensis* Fisch. [18]. The distinctive feature lies in the rare trans-dihydroxylation at C-24/C-25, particularly the unusual occurrence of the C-24 hydroxyl moiety. The final mogrosides are formed through different glucosylation activities in the C3-OH and C24-OH positions via UGT (Figure 1). Previous studies have illustrated the biosynthetic pathway of mogrosides and identified almost all key enzymes involved in biosynthesis except for squalene epoxidase (SQE) [13,16,19,20,21,22,23]. Unlike conventional triterpenoids derived from the universal precursor 2,3-oxidosqualene (SQO), mogrol is believed to be derived from 2,3;22,23-diepoxysqualene (SDO) as its linear precursor. Itkin et al. demonstrated that SDO is cyclized by cucurbitadienol synthase (CS) to 24,25-epoxy cucurbitadienol and subsequently hydrolyzed by epoxide hydrolase (EPH) to yield mogrol with the distinctive C24-OH position. However, the specific SQE that catalyzes the generation of SDO in *S. grosvenorii* has not yet been identified, and the unique enzymatic mechanism of this reaction is still unclear. It is hypothesized that among the *SgSQE* isoforms, one or more *Sg*SQEs may have the ability to catalyze two-step epoxidation, converting squalene to SDO, thereby contributing specifically to mogroside biosynthesis. This study aims to functionally characterize the squalene epoxidases of *S. grosvenorii* and investigate their involvement in mogrosides biosynthesis.

## 2. Results

### 2.1. Cloning and Sequence Analysis of SQEs in S. grosvenorii

**Figure 2:**
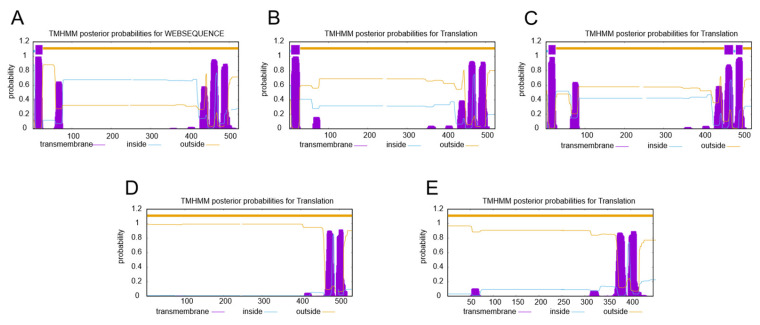
Transmembrane domain prediction for *Sg*SQE isoforms via TMHMM Server. (**A**): *Sg*SQE1 (**B**): *Sg*SQE2 (**C**): *Sg*SQE3 (**D**): *Sg*SQE4 (**E**): *Sg*SQE5.

In this study, a total of seven full-length *SgSQE*s were obtained for functional analysis. *SgSQE1* and *SgSQE2* were cloned from *S. grosvenorii* cDNA previously; *SgSQE3*, *SgSQE4*, *SgSQE5*, *SgSQE6*, and *SgSQE7* were obtained through gene synthesis. The ORFs of the 7 *SgSQE* genes were 1575, 1575, 1572, 1605, 1350, 246, and 927bp, which were predicted to encode 524, 524, 523, 534, 449, 81, and 308 amino acids, respectively. All predicted *Sg*SQE proteins except *Sg*SQE6 had a conserved domain identified as squalene monooxygenase, while *Sg*SQE7 just contained a Rossmann-fold NAD(P)(+)-binding domain. The TMHMM analysis suggested that both *Sg*SQE1 and *Sg*SQE2 had only one N-terminal transmembrane domain; *Sg*SQE3 had two transmembrane domains with an N-terminal and C-terminal, respectively; and *Sg*SQE4 and *Sg*SQE5 were located outside the membrane without obvious transmembrane domains (Figure 2).

**Figure 3:**
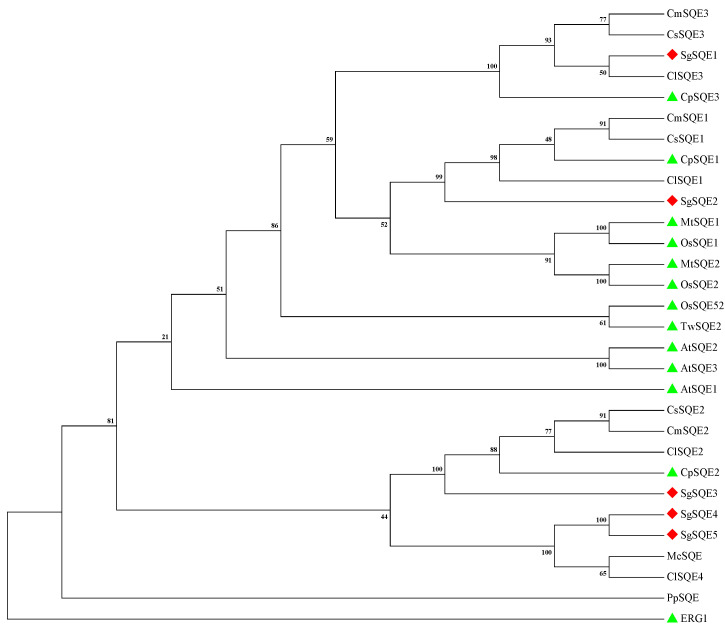
Phylogenetic analysis of *Sg*SQE and other SQEs from various species by MEGA11 using the neighbor-joining method with 1000 bootstraps. *At*SQE1: NM119938 *Arabidopsis thaliana* (L.) Heynh., *At*SQE2: NM127848 *A. thaliana* (L.) Heynh., *Mt*SQE1: AJ430608 *M. truncatula* Gaertn., *Mt*SQE2: AJ430609 *M. truncatula* Gaertn., *Os*SQE1: AJ430608 *O. spinosa* L., *Os*SQE2: AJ430609 *O. spinosa* L., *Cp*SQE1: MH243446 *Cucurbita pepo* L., *Cp*SQE2: MH243447 *C. pepo* L., *Cp*SQE3: MH243445 *C. pepo* L., *Cl*SQE1: Cla006490 *Citrullus lanatus* (Thunb.) Matsum. and Nakai, *Cl*SQE2: Cla001251 *C. lanatus* (Thunb.) Matsum. and Nakai, *Cl*SQE3: Cla020903 *C. lanatus* (Thunb.) Matsum. and Nakai, *Cs*SQE1: XM004136871 *C. sativus* L., *Cs*SQE2: XM004141255 *C. sativus* L., *Cs*SQE3: XM004142859 *C. sativus* L., *Cm*SQE1: XM008457301 *Cucumis melo* L., *Cm*SQE2: XM017045860 *C. melo* L.; *Cm*SQE3: XM008446295 *C. melo* L.; *Mc*SQE: XP_022149174.1 *Momordica charantia* L., *Tw*SQE2: MG717396.1 *Tripterygium wilfordii* Hook. f., *Os*SQE52: *Oryza sativa* L., *Pp*SQE: XM_001781268 *Physcomitrella patens* (Hedw.) Bruch and Schimp., ERG1: YGR175C *Saccharomyces cerevisiae*. All the amino acid sequences except *Os*SQE52 [25] were retrieved from GenBank with accession numbers. The green triangle represents functional SQEs, while the red diamond represents *Sg*SQE isoforms.

The phylogenetic analysis of *Sg*SQEs revealed four distinct clades in Cucurbitaceae, with *Sg*SQE1 clustering into Clade III, *Sg*SQE2 clustering into Clade I, *Sg*SQE3 clustering into Clade II, and *Sg*SQE4 and *Sg*SQE5 clustering into Clade IV (Figure 3). Compared with SQE proteins that have been identified, *Sg*SQE1, *Sg*SQE2, and *Sg*SQE3 are evolutionarily closer to SQEs of *Ononis spinosa* L., *Medicago truncatula* Gaertn., and *Morus alba* L. *Sg*SQE4 was closely related to the SQE of *Morella rubra* Lour. Furthermore, the results of multiple sequence analysis performed using DNAMAN 8.0.8 software showed that *Sg*SQE1 exhibited 77% and 75.6% identity to *Os*SE1 (AUD09558) and *Os*SE2 (AUD09559), respectively. *Sg*SQE2 exhibited 77% and 75.3% identity to *Os*SE1 and *Os*SE2, respectively. *Os*SQE1 and *Os*SQE2 were identified to accumulate SDO in tobacco [24]. These results indicated that *Sg*SQE1 and *Sg*SQE2 may also catalyze the formation of SDO from squalene.

**Figure 4:**
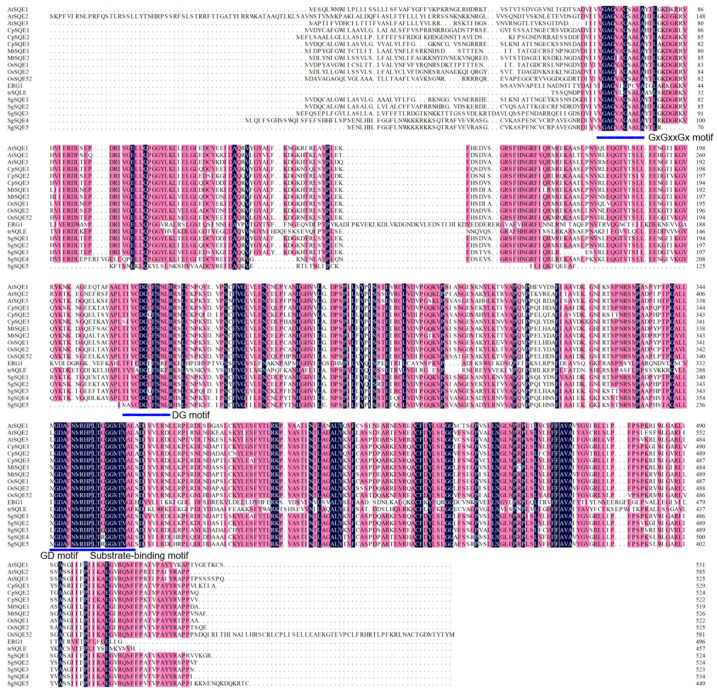
Multi-alignment of functional SQEs from other species and *Sg*SQEs. The underlines in blue represent key motifs. trSQLE was human SQLE with truncation of N-terminal.

The multiple alignment of four *Sg*SQEs and SQE proteins from other plants showed that *Sg*SQE1-4 all contain an FAD/NAD(P)-binding domain and a substrate-binding domain, which are highly conserved and essential for the catalytic activity [26]. Interestingly, there was a difference in the DG motif of SgSQE3, where Ala385, other than the common Ser385, was present in *Sg*SQE3; moreover, Arg367 and Arg269, other than the common Gly, were present in *Sg*SQE4 and *Sg*SQE5, which may influence the substrate binding (Figure 4).

### 2.2. Functional Identification in ∆erg1 Saccharomyces cerevisiae

The genetically modified strain SZ08 was generated by the following: (i) chromosomal integration of the exogenous SQE gene, followed by (ii) targeted knockout of the endogenous ERG1.

**Figure 5:**
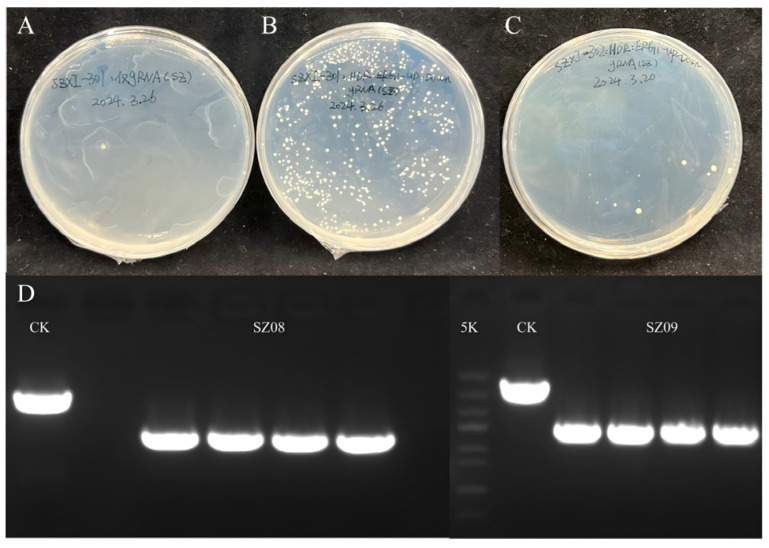
The modified strain SZ08 and strain SZ09 on SC-URA medium, and the colony PCR result. (**A**): Control with *ERG1*-targeted gRNA, (**B**): the strain SZ08 was generated with integration of the exogenous *SgSQE1*, followed by knockout of the endogenous *ERG1*, (**C**): SZ09 was generated with integration of the exogenous *SgSQE2*, followed by knockout of the endogenous *ERG1*, (**D**): gel electrophoresis of colony PCR, CK represents control only with *ERG1*-targeted gRNA.

To elucidate the function of the *SgSQE* genes, the exogenous *SgSQE* was introduced into a yeast chromosome, followed by the knockout of the endogenous gene *erg1* using the ideal gRNA5. As observed in Figure 5A–C, the modified strains with only *SgSQE1* or *SgSQE2* were capable of normal growth and were designated as SZ08 and SZ09 after positive colony PCR (Figure 5D), demonstrating that both *Sg*SQE1 and *Sg*SQE2 exhibit squalene epoxidase (SQE) activity.

**Figure 6:**
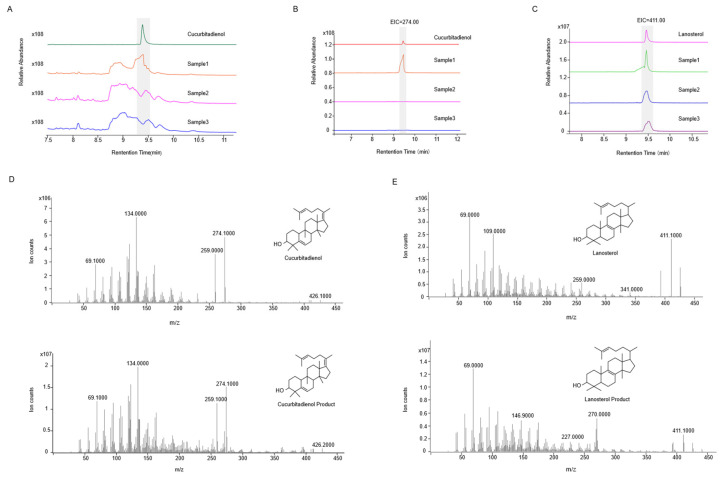
Gas chromatography and mass spectrometry (MS) of cucurbitenol and lanosterol from strains THY02, SZ08, and SZ09, harboring the pESC-SgCS-URA plasmid. Sample1, Sample2, and Sample3 were strain THY02, strain SZ08, and strain SZ09, harboring the pESC-*SgCS*-URA plasmid. (**A**): Total ion chromatograms (TICs) of standards and samples of cucurbitenol, (**B**): extraction ion chromatogram (EIC) of characteristic fragment ions of cucurbitenol with a mass/charge ratio (*m*/*z*) of 274, (**C**): EIC of characteristic fragment ions of lanosterol with *m*/*z* of 411, (**D**): MS of the standard and product of cucurbitenol, (**E**): MS of lanosterol standard and lanosterol product.

**Figure 7:**
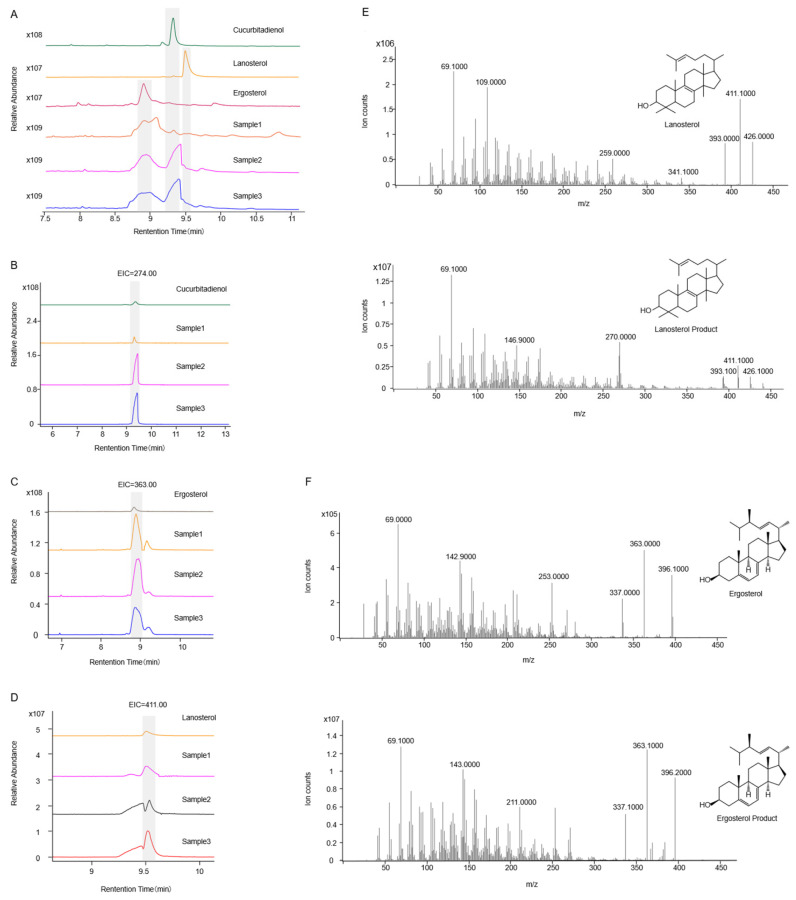
The yeast extracts detected by GC-MS after *SQE*/*ERG1* overexpression. (**A**): TIC of standard and samples of cucurbitenol, (**B**): EIC of characteristic fragment ions of cucurbitenol (*m*/*z* = 274), (**C**): EIC of characteristic fragment ions of cucurbitenol (*m*/*z* = 411), (**D**): MS of the standard and product of cucurbitenol, (**E**): MS of lanosterol standard and lanosterol product, (**F**): MS of ergosterol standard and ergosterol product. Sample1 was the strain THY02 harboring the pESC-ERG1-SgCS-URA plasmid, Sample2 was the strain SZ08 harboring the pESC-SgSQE1-SgCS-URA plasmid, Sample3 was the strain SZ09 harboring the pESC-SgSQE2-SgCS-URA plasmid.

To better characterize the catalytic products of *Sg*SQE, we initially attempted to detect squalene epoxide. However, due to its low abundance and challenges in enrichment and detection, the cyclase *Sg*CS, which utilizes squalene epoxide as a substrate, was introduced to facilitate the detection of more readily accumulated cyclized products. When *SgCS* was solely introduced into strain THY02 and the modified strains, lanosterol was detected in all strains, whereas cucurbitadienol was absent from the modified strains SZ08 and SZ09 compared with THY02, which contained a single copy of *ERG1* (Figure 6). However, upon the co-expression of *SgCS* with an extra copy of *SgSQE1*/*SgSQE2/ERG1* in the corresponding strainSZ08, SZ09, andTHY02, both lanosterol and cucurbitadienol were identified through GC-MS analysis (Figure 7). These results indicated that the squalene epoxide generated by *Sg*SQEs was preferentially utilized for lanosterol biosynthesis at low concentrations, a process essential for yeast growth, and was channeled toward secondary metabolic pathways only when its accumulation reached a certain concentration.

**Figure 8:**
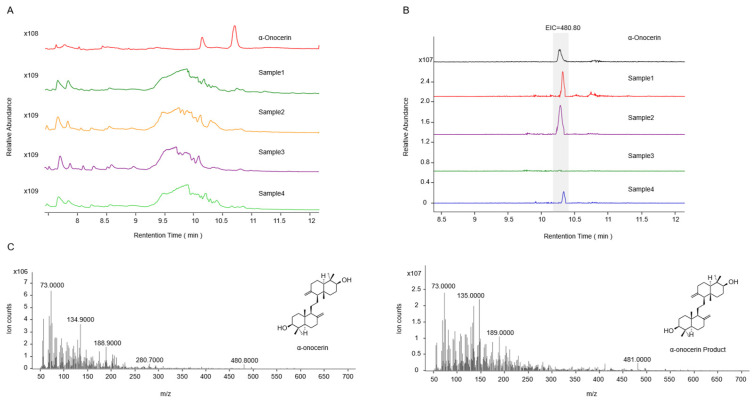
Gas chromatography and MS spectra of α-onocerin from the modified strains. Sample1 was the strain SZ12 harboring the pESC-*ERG1*-*OsONS1*-URA plasmid, Sample2 was the strainSZ15 harboring the pESC-*OsSQE1*-*OsONS1*-URA plasmid, Sample3 was the strain SZ13 harboring the pESC-*SgSQE1*-*OsONS1*-URA plasmid, Sample4 was the strain SZ14 harboring the pESC-*SgSQE2*-*OsONS1*-URA plasmid. (**A**): TIC of standard and samples of α-onocerin; (**B**), EIC of characteristic fragment ions of α-onocerin (*m*/*z* = 480.80); (**C**):MS of the standard and product of α-onocerin.

Although cucurbitadienol synthase is capable of catalyzing the formation of cucurbitadienol and 24,25-epoxycucurbitadienol from SQO and SDO, respectively, the absence of a 24,25-epoxycucurbitadienol reference standard hindered the experimental analysis. To further determine whether SDO was produced by *Sg*SQEs, *pERG7* was substituted with a weak promoter, *pHXT1*, first in strains SZ08, SZ09, and THY02, resulting in strains SZ13, SZ14, and SZ12. In addition, for a positive control, strain SZ15 was obtained with the introduction of *OsSQE1* and knockout of *erg1*, along with the substitution of *pERG7* to *pHXT1*. Subsequently, *SgCS* was replaced with the functional *OsONS1*, catalyzing exclusively SDO into α-onocerin [24], and finally, α-onocerin was detected in strain SZ14 after an 8-day fermentation period (Figure 8). This study provides mechanistic insights into the functional divergence of SQE enzymes and their regulatory roles in primary and secondary metabolism.

### 2.3. Subcellular Localization Analysis of SgSQEs

**Figure 9:**
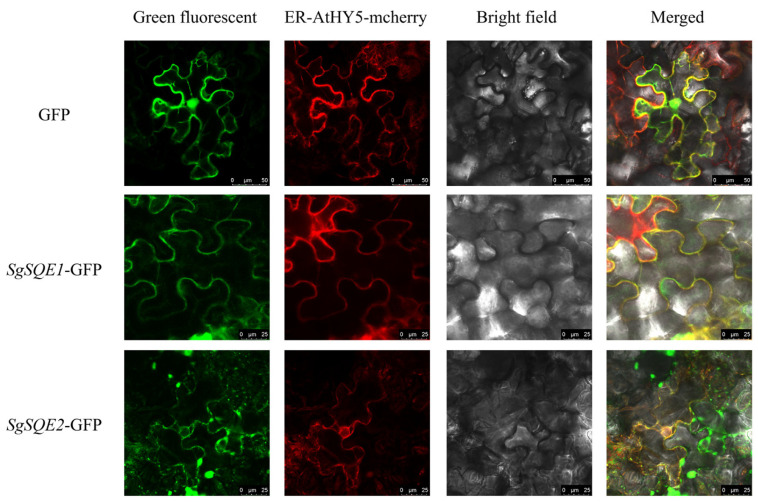
Subcellular localization of *Sg*SQE1 and *Sg*SQE2. Subcellular localization of *Sg*SQE-GFP fusion proteins in tobacco epidermal cells. GFP was selected as control, and ER-mcherry was used as a positive control. The photographs were taken in the green channel (GFP fluorescence), red channel (mCherry fluorescence), combination of green and red channel, and bright channel.

Squalene epoxylases are generally believed to be localized in the endoplasmic reticulum (ER). The DeepLoc-2.1 online software [27] was utilized for prediction, and both *Sg*SQE1 and *Sg*SQE2 were most likely to be localized in the ER. To define the subcellular localization of *Sg*SQE1 and *Sg*SQE2, the resulting *Sg*SQE-eGFP fusion proteins were transiently injected into tobacco epidermal cells. As shown in Figure 9, the fluorescence signals of *Sg*SQE1-eGFP and *Sg*SQE2-eGFP were almost identical to those of the ER-marker protein (AtHY05-mcherry). This result demonstrated that the catalytic reactions by *Sg*SQE1 and *Sg*SQE2 were distributed in the ER, which was consistent with their localization predictions.

### 2.4. Expression Patterns of SgSQE1 and SgSQE2 in S. grosvenorii

**Figure 10:**
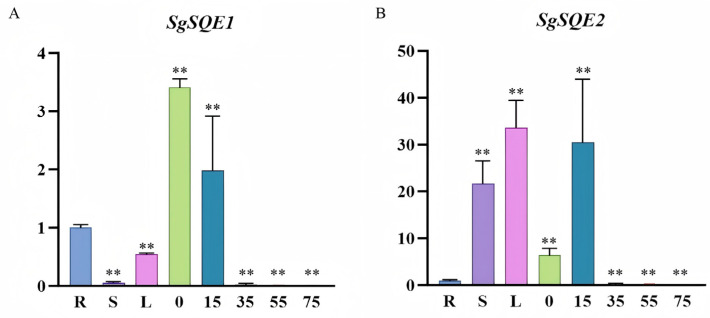
Tissue expression patterns of the functional *SgSQE1* and *SgSQE2* genes. (**A**): The expression levels of *SgSQE1* in roots, stems, leaves, and fruits at different developmental stages. (**B**): The expression levels of *SgSQE2* in roots, stems, leaves, and fruits at different developmental stages. R: root, S: stem, L: leaf, 0/15/35/55/75: represent the number of days the fruit grows. The relative expression levels of *SgSQE1* and *SgSQE2* are calculated using the 2^−ΔΔCt^ method, with the value in roots as 1. All data indicate means ±SD from three replicates. Statistical significance is denoted by ‘**’ (*p* < 0.01).

To investigate the roles of functional *SgSQE1* and *SgSQE2* in *S. grosvenorii*, qRT-PCR was conducted to determine the expression patterns in different organs, including roots, stems, leaves, and fruits. Both *SgSQE* and *SgSQE2* were constitutively expressed in these organs, with high expression in fruits. Furthermore, the expression levels of *SgSQE1* and *SgSQE2* in fruits of different growth periods (0 d, 15 d, 35 d, 55 d, and 75 d) were further investigated. *SgSQE1* exhibited the highest level at 0d and dramatically decreased during the fruit development (Figure 10A). The expression level of *SgSQE2* increased sharply with the growth and development of fruit, and reached the maximum at 15 d, and then dropped sharply at 30 d (Figure 10B). These results suggested that the expression pattern of *SgSQE2* in fruits was consistent with the only one *SgCS* gene within 75 days post-anthesis, and indicated that *SgSQE2* likely plays a key role in mogroside biosynthesis.

### 2.5. Molecular Docking Analysis and Determination of Key Residues of SgSQE

**Figure 11:**
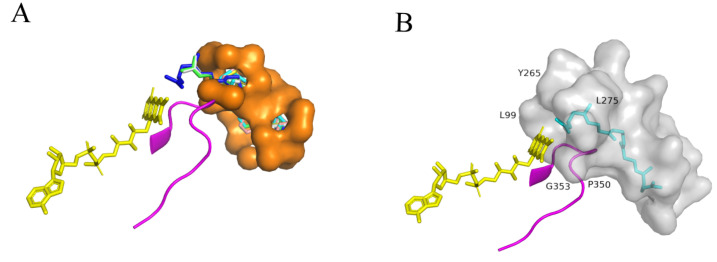
The structure of *Sg*SQE1-FAD-NADPH complex with substrate squalene. ((**A**): Prediction of the substrate binding pocket of SgSQE1-FAD-NADPH by PrankWeb. Yellow represents FAD Sticks model, magenta represents the Cartoon model of the ‘NMRHPLTGGG’ motif, orange represents the Surface model of substrate binding pocket, and the colored molecules in the pocket represent the Sticks model of 7 squalene molecules after docking; (**B**): Docking Results of *Sg*SQE1-FADPH Complex with the substrate squalene. Gray represents the Surface model of amino acid residues within the 4 Å range of substrate squalene, while Cyan represents the Sticks model of squalene after docking).

**Figure 12:**
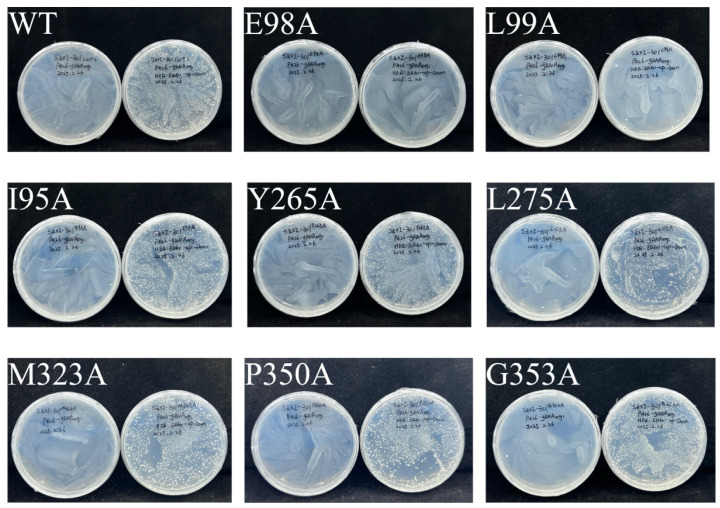
Growth of SZ08 mutant on SC-Ura medium.

To better understand the key residues for squalene epoxidase function, molecular docking was performed using the predicted *Sg*SQE protein structure (along with the cofactors FAD and NADPH) and the substrate squalene via AutoDock Vina. Given the 83.56% identity between *Sg*SQE1 and *Sg*SQE2 amino acids with major variations during the N-terminal transmembrane domain, *Sg*SQE1 was chosen for structure prediction using AlphaFold v2.0 [28]. The tunnel of squalene predicted through PrankWeb online was located in the pocket and consisted of residues within a 4 Å radius of the substrate, as visualized in the PyMol viewer docking result (Figure 11). The conserved motif ‘NMRHPLTGGG’ was located close to the FAD-binding domain and the potential binding domain of squalene in rat SQE, as previously reported [29]. This motif was predicted as a loop parallel to the isoalloxazine ring of FAD; hence, the grid box was selected, taking this loop at an interface between FAD and the potential squalene tunnel. Combined with the multiple sequence alignment, eight residues near the entrance of the pocket and adjacent to FAD were ultimately selected for mutagenesis with alanine scanning. These eight residues were I95, E98, L99, Y265, L275, M323, P350, and G353, respectively. The mutants were obtained with the same method as strains SZ08 and SZ09 by introducing exogenous *SQE^mutant^* and then knocking out endogenous *erg1*. As a result, the E98A and L99A mutants failed to grow, indicating that E98 and L99 were key residues for functional squalene epoxidase (Figure 12).

**Figure 13:**
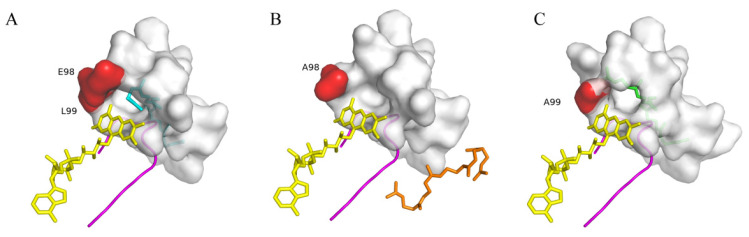
Docking Results of SgSQE1^mutant^-FAD-NADPH Complex with squalene. ((**A**): Docking Results of SgSQE1-FAD-NADPH WT Complex with squalene. Yellow represents FAD Sticks model, magenta represents the Cartoon model of the ‘NMRHPLTGGG’ motif, Gray represents the Surface model of amino acid residues within the 4 Å range of substrate squalene, cyan, orange, and green represent the Sticks model of squalene after docking; (**B**): Docking Results of *Sg*SQE1^E98A^-FAD-NADPH WT Complex with squalene; (**C**): Docking Results of *Sg*SQE1^L99A^-FAD-NADPH WT Complex with squalene).

Furthermore, we performed docking analysis using *Sg*SQE1^E98A^ and *Sg*SQE1^L99A^ mutants with squalene. In the model of WT *Sg*SQE1 (Figure 13A), squalene was deeply embedded within a narrow hydrophobic tunnel featuring the only opening toward the isoalloxazine ring of FAD, which was highly similar to the human SQLE structure. Interestingly, E98 and L99 were located in the entrance of the squalene pocket and near the isoalloxazine ring. The substitution with nonpolar alanine for the negatively charged E98 (E98A) destabilized FAD binding, subsequently leading to squalene collapse from the narrow tunnel (Figure 13B). This finding demonstrates that E98, located at the pocket entrance, plays a critical role in maintaining FAD-binding integrity. The L99A mutation, which substitutes leucine with a smaller alanine residue, distorted squalene’s conformation and prevented its reactive double bond from aligning with FAD for oxygenation (Figure 13C). This result indicated that L99 may stabilize squalene in a catalytically competent manner.

## 3. Discussion

Squalene epoxidase ([EC1.14.99.1]), also known as squalene monooxygenase, catalyzes the epoxidation of squalene and is the key rate-limiting enzyme in triterpene and sterols biosynthesis. While SQEs have been extensively characterized in humans [30,31,32], animals [33], and yeast [34,35,36,37], research on plant SQEs remains relatively limited, particularly regarding their roles in secondary metabolism. SQEs in plants were less mechanistically understood compared to other key enzymes such as cytochrome P450s or oxidosqualene cyclases (OSCs). Most of the identified SQEs were confirmed predominantly through complementation assays in the *erg1*-deficient *S. cerevisiae* strain KLN1 [38,39], strain JP064 [40], or strain RXY6 [41]. Although widely employed, this approach had some technical limitations. For example, the conventional *erg1*-deficient mutants require anaerobic cultivation with exogenous ergosterol supplementation. Furthermore, heterologous *SQE*s were introduced via episomal plasmids, which easily suffer from plasmid loss or unstable expression. In this study, we developed an effective method for *SQE* functional characterization, which involves the genomic integration of heterologous *SQE* genes followed by the knockout of the endogenous *ERG1* gene, circumventing the stringent culture requirements associated with the K1N1 strain. However, further exploration of their catalytic products remains scarce. Only a few reports detected products catalyzed by plant SQE. Han et al. identified *PgSE1* and *PgSE2* producing SQO via a functional complementation test using the *erg1* mutant in yeast [42]. As for cucurbit SQEs, Zhang found that prokaryotic expression of *HmSE* yielded SQO [43]; Chen et al. heterologously expressed three *HcSE* isoforms in *E. coli* and two *HcSEs* produced SQO but no signal of SDO, while *HcSE3* failed to express in BL21(DE3) [44]. However, three *CpSQE*s showed dual SQO and SDO production in an *erg1 erg7* mutant, compared with the previously characterized cucurbit SQEs [40]. When the *erg1 erg7* mutant was expressed, *AtSQE1* and *AtSQE3* could convert squalene to SQO and SDO, while *AtSQE2* only produced SQO, indicating *Arabidopsis* SQE enzymes had distinct substrate preferences [41]. All the products by SQE were detected by GC-MS. In this study, we failed to measure the SQO or SDO due to the impurity of the reference standard we purchased and the low levels in organisms. The commercially available reference standards exhibited multiple peaks with similar ion fragments, and the low endogenous levels of SQO or SDO were easily masked by dominant yeast sterols. Thus, we analyzed the cyclized products by co-expressing exogenous downstream OSC, indirectly confirming in vivo epoxidation. α-onocerin is cyclized exclusively from SDO by *Os*ONS as previously reported by Rowan and Almeida [24,45]. The products catalyzed by *Os*ONS were exclusively derived from the introduced pathway, with no detectable endogenous analogs in control yeast strains, therefore, *OsONS1* was introduced in this study. We found that SQO was maintained at a relatively low level in vivo, and was preferentially channeled to sterol biosynthesis for basic growth and then to triterpenoid scaffold formation. When SQO accumulates to some extent, SQO causes further conversion by SQE to SDO to occur. Therefore, strain SZ14 was capable of producing α-onocerin using SDO catalyzed by *Sg*SQE2 as a substrate. Previous studies have demonstrated that SQEs from mammals or some plants can synthesize SDO only after the treatment of OSC inhibition [41]. Whether *Sg*SQE1 can produce SDO under specific conditions requires further investigation.

Unlike yeast and mammals that typically possess a single *SQE* gene, plants often harbor multiple *SQE* copies with tissue-specific expression patterns [41,46,47]. There were six SQE homologs in *Arabidopsis*; *SQE1* and *SQE3* appear to be widely expressed, while *SQE2* and *SQE4* show low-level expression patterns. *SQE3* shows consistently high expression across developmental stages, suggesting its predominant role in basal sterol biosynthesis [41]. In *Hemsleya macrosperma*, *HmSE1* transcripts significantly exceed those of *HmSE2* and *HmSE3*, and increased significantly with MeJA treatments, which specifically participate in cucurbitacin biosynthesis [43]. SQE isoforms exhibit differential expression patterns and MeJA responsiveness across plant species, as also observed in *M. truncatula* Gaertn., *Panax ginseng* C. A. Mey., and *C. pepo* L. The silencing of *PgSQE1* enhanced *PgSQE2* expression levels and stimulated phytosterol production, suggesting *PgSQE2* gene positively regulates sterol production [46]. In this study, *SgSQE2* exhibited a similar expression profile with mogroside accumulation in *S. grosvenorii* fruits. This finding suggests that distinct SQE isoforms may differentially contribute to sterol and triterpenoid biosynthesis in plants, including *S. grosvenorii* [41,44].

SQE typically requires the cofactors FAD and NADPH for catalytic activity [48]. This reaction depends on NADPH-cytochrome P450 reductase (CPR) [49], which mediates the transfer of an epoxide group to the carbon–carbon double bond of squalene [32]. Following flavin reduction, NADP+ is promptly released. According to multi-sequence alignment analysis, several conserved features containing the GxGxxGx motif, GD motif, and DG motif are characteristic of flavin monooxygenases [50]. The NMRHPLTGGG in the GD motif has been reported to be the binding site of FAD and squalene [29]; in this study, we conducted site-directed mutagenesis for *Sg*SQE1 and screened two key residues, E98 and L99, close to the GD motif. Our docking analysis revealed that both squalene and FAD bind to the opposite sides of the GD motif, and squalene was in an elongated hydrophobic pocket with a single opening toward the GD motif and the isoalloxazine ring of FAD. Following the epoxidation of squalene to SQO, the product dissociates from the pocket after FAD reduction. Interestingly, SDO formation requires SQO to re-enter the pocket in a distinct orientation with the epoxide group distal to the pocket entrance, enabling the second epoxidation of SQO to generate SDO. This dual-positioning mechanism may explain why SDO production only occurs when SQO accumulates to a certain concentration. These findings could provide evidence for the earlier reports of SDO accumulation through OSC inhibition, because the SQO was the same substrate for the second epoxidation by SQE and cyclization by OSC. In addition, the structural plasticity of the FAD-binding domain appears crucial for this dual catalytic capability. The precise molecular mechanism underlying the epoxidation activity remains unclear. Future cryo-EM studies to resolve the ternary complexes of SQE with cofactors and substrates would be invaluable for elucidating the structural basis of this process.

As a rate-limiting enzyme, SQE represents a crucial functional module for both sterol and triterpenoid production. In synthetic biology approaches for triterpenoid biosynthesis, increasing the *ERG1* copy number remains the most prevalent strategy for flux enhancement. However, reports on improving SQE catalytic activity via mutagenesis are few. Li et al. compared different SQEs and conducted alanine scanning and saturation mutagenesis for *Os*SQE52, and the β-amyrin level was increased 1.54-fold [25]. Yin et al. achieved a 64% increase in ergosterol using V249/L343 double mutants [36]. In the future, the mutagenesis of various amino acid residues combined with SQE protein structure, compartmentalization engineering, SQE/OSC, or SQE/CPR protein–protein interaction may be an effective strategy to enhance SQE catalytic efficiency and metabolic flux.

## 4. Conclusions

In conclusion, this study represents the first systematic investigation of SQE function derived from *S. grosvenorii*. Among seven genes annotated as SQE from *S. grosvenorii*, we identified two isoforms (*SgSQE1* and *SgSQE2*) exhibiting squalene epoxidase function. This was the first report for *Sg*SQE2 capable of catalyzing dual epoxidation reactions, which completed the mogrosides biosynthetic pathway. We developed a genomically engineered strategy for heterologous SQE characterization, which could overcome limitations of the conventional *erg1*-deficient mutants and enhance operational feasibility without special growth requirements. The alanine scanning results suggested that E98 and L99 were key residues for SQE, and the catalytic mechanism was proposed through docking analysis, which could explain the two-step continuous catalytic reactions. Moreover, the gene expression patterns and the subcellular localizations in the ER provide more information for functional *Sg*SQEs. Overall, this study may not only fill the gap in the examination of SQE catalytic reaction for mogrosides biosynthesis, but also provide gene resources and feasible ideas for triterpene metabolic engineering.

## 5. Materials and Methods

### 5.1. Plant Materials and Plasmids

*S. grosvenorii* was grown in Guilin Yiyuansheng Modern Biotechnology Co., Ltd. (Guilin, China), Guangxi Zhuang Autonomous Region. Samples of fruits with different growth periods, leaves, and stems were collected from three individual plants; all tissues were frozen immediately in liquid nitrogen and stored at –80 °C for gene cloning and gene quantification. *SgSQE*1 and *SgSQE*2 genes were preserved as the recombinant plasmids pBlunt-*SQE*1 and pBlunt-*SQE*2 in our laboratory.

### 5.2. Gene Cloning and Gene Synthesis

Two full-length *SgSQE*s were previously annotated from our transcriptome data, of which *SgSQE*1 and *SgSQE*2 have been successfully cloned from cDNA and stored in the pEASY-Blunt vector previously. In addition, five more *SgSQE*s were annotated in the *S. grosvenorii* genome database (data not published) combined with the report by Itkin [20], and were obtained in the pUC57 vector through gene synthesis by RuiBiotech, which were named *SgSQE3*, *SgSQE4*, *SgSQE*5, *SgSQE*6, and *SgSQE*7. All sequences of the seven *SgSQE* genes were listed in Supplementary Information Table S1. The primers for *SgSQE*s cloning were designed using Primer Premier 5.0 software and are listed in Supplementary Information Table S2.

### 5.3. Bioinformatic Analysis of SgSQEs

The open reading frames (ORFs) of SQEs were identified online using the NCBI tool ORF Finder (https://www.ncbi.nlm.nih.gov/orffinder/ accessed on 15 July 2022) and conserved domains were predicted (https://www.ncbi.nlm.nih.gov/Structure/cdd/wrpsb.cgi/ accessed on 15 July 2022). The online software TMHMM (http://www.cbs.dtu.dk/services/TMHMM/ accessed on 15 July 2022) was used to predict the transmembrane structure of the proteins. To visualize the conserved motifs and compare the homology between sequences, multiple sequence alignments were performed using the software DNAMAN (version 9.0). We downloaded the amino acid sequences of SQEs of other species from the National Center for Biotechnology Information (NCBI) database and aligned them using ClustalW; then, a neighbor-joining tree was built using MEGA7 software [51], and the number of bootstrap iterations was 1000.

### 5.4. Functional Characterization of SgSQEs in S. cerevisiae

Considering *erg1* knockout in *S. cerevisiae* is lethal, the exogenous *SgSQE*s were first integrated into the X1-3 site, and then *ERG1* was knocked out using CRISPR-cas9 editing.

#### 5.4.1. Heterologous Expression of the SgSQE Gene in *S. cerevisiae*

The strain THY02 expressing Cas9 with Gal80 knockout was employed for the integration of *SgSQE*s into the XI-3 site. The guide RNA (gRNA) targeted at the XI-3 site with the sequence of ‘ATATGTCTCTAATTTTGGAA’ was recombined into the p426 vector, which was stored in our lab. The TEF1 promoter and CYC1 terminator, amplified from the *S. cerevisiae* genome, were fused with *SgSQEs* via overlap-PCR to generate the *P_TEF1_*-*SgSQE1*-*T_CYC1_* expression cassette, which was subsequently combined with upstream and downstream homologous repair (HR) fragments through another overlap-PCR reaction, yielding the final integration fragment (designated as HR XI-3-*SgSQE*s) targeting the XI-3 genomic locus. The gene sequences, including promoters, terminators, integration sites, and upstream and downstream HRs, were searched from the Saccharomyces Genome Database (SGD, https://www.yeastgenome.org) and provided in Table S1. All the primers for genome editing at the XI-3 site were designed using Benchling online (https://www.benchling.com/ accessed on 1 January 2023) and deposited in Table S2.

The HR XI-3-*SgSQE*s fragments were introduced into strain THY02 together with gRNA using the lithium acetate transformation method, followed by selection on an SD-Ura plate. After incubating at 30 °C for 48 h, colonies were randomly picked and validated with specific primers using PCR and sequencing. To avoid the cleavage of this target, the gRNA5-p426 plasmid was removed. All of the modified strains above were cultured on SC full nutrient solid medium containing 1 mg/mL 5-FOA for 2–3 days. The new colonies were coated on an SD-Ura and YPD solid medium, respectively, and the ones that succeeded in growing on YPD but failed to grow on SC-Ura were considered successful gRNA removal. The positive colony with *SgSQE*1 was designated as SZ01, and the other right colonies with *SgSQE*2~*SgSQE*7 were designated as SZ02~SZ07, respectively.

#### 5.4.2. Knockout of *erg1*

The gRNA was searched against the target *ERG1* gene sequence using an online tool (http://chopchop.cbu.uib.no/ accessed on 1 November 2023), which was a 20-mer sequence together with an NGG protospacer-adjacent motif (PAM) sequence (N 20 NGG). The top five specific gRNAs (Table 1) were obtained via PCR and ligated into the plasmid p426, and then the resulting p426-gRNAs were introduced into *S. cerevisiae* strain THY02 and screened with SD-Ura solid medium at 30 °C for 48 h; finally, the one with the fewest colonies was selected as the ideal gRNA.

To knockout the entire *ERG1* gene in yeast strain THY02, the HR donors were flanked at the ends of *ERG1*-ORF, of which the upstream HR (581 bp) and downstream HR (577 bp) shared 50 bp homology arms. Overlap PCR was performed to ligate the upstream HR and downstream HR directly, and the generated HR was transformed into strains SZ01~SZ07 as above. After two days, single colonies were selected and further checked using PCR; the positive colonies after gRNA removal were designated as SZ08 and SZ09. In addition, strain SZ10 with *OsSQE1* insertion and *erg1* knockout was genetically obtained as above. The primers used for the knockout ERG1 gene are listed in Supplementary Information Table S3.

#### 5.4.3. Construction of *SQE* Expression Vectors

To construct expression vectors, seamless splicing primers containing the coupling sequence were designed (Table 1). The linearized vector pESC-*SgCS*-Ura was generated through segmented PCR amplification after DMT digestion and was linked with *SgSQEs*/*ERG1* PCR products at 50 °C for 15 min using the pEASY^®^-UniSeamless Cloning and Assembly Kit (TransGen Biotech, Beijing, China), yielding the vectors pESC-*SgSQE1*-*SgCS*-Ura, pESC-*SgSQE2*-*SgCS*-Ura, and pESC-*ERG1*-*SgCS*-Ura, respectively. The recombinant vectors pESC-*SgSQE1*-*OsONS1*-Ura, pESC-*SgSQE2*-*OsONS1*-Ura, pESC-*OsSQE1*-*OsONS1*-Ura, and pESC-*ERG1*-*OsONS1*-Ura were obtained using the same method, and the target gene *OsONS1* was cloned using pUC57-*OsONS1* as the template. The primers used for constructing the SQE overexpression vector and *OsONS1*-SQE overexpression vector are listed in Supplementary Information Tables S6 and S7.

#### 5.4.4. Replacement of ERG7 Promoter with Weak Promoter pHXT1

To reduce flux to lanosterol biosynthesis by ERG7, *pHXT1* with 50 bp homology arms was amplified and inserted into the strains THY02, SZ08, SZ09, and SZ10 with p426-gRNA*_pERG7_* as gRNA using the through lithium acetate transformation method. The positive colonies after gRNA removal were designated as SZ12, SZ13, SZ14, and SZ15. The primers used for replacing the ERG7 promoter are listed in Supplementary Information Table S4.

#### 5.4.5. Yeast Fermentation and Product Detection Using GC-MS

The constructed over-expression vectors (pESC-*SgSQE1*-*SgCS*-Ura, pESC-*SgSQE2*-*SgCS*-Ura, etc.) were introduced into the corresponding SZ08, SZ09, etc., using the Frozen-EZ Yeast Transformation II Kit (ZYMO RESEARCH, Irvine, CA, USA). The PCR-positive colonies were further cultured on SC-Ura and supplemented with glucose (2%). The transformants were induced using 2% galactose, collected, and lysed using 20% KOH and 50% EtOH as reported by [47]. The products were extracted using an equal volume of hexane three times and detected on an Agilent 7890B gas chromatography tandem 7000C GC/MS mass spectrometer (splitless, injector temperature: 250 °C) with a DB-5ms (15 m × 250 μm × 0.1 μm) capillary column. The ion trap temperature was 250 °C. The electron energy was 70 eV. Spectra were recorded in the range of 10–700 *m*/*z*.

For α-onocerin detection, 1 μL of the concentrated organic phase was then injected under a He flow rate of 1 mL min^−1^ with a temperature program of 1 min at 50 °C, followed by a gradient from 50 to 270 °C at 50 °C min^−1^ and then to 305 °C at 20 °C min^−1^ with an 11.85 min hold. As for α-onocerin, the initial temperature of the column temperature box was set to 40 °C, followed by a gradient from 50 to 270 °C at 50 °C min^−1^ and then to 320 °C at 9 °C min^−1^ with a 2 min hold.

### 5.5. Subcellular Localization

The possible subcellular localization of *Sg*SQEs was predicted through an online tool DeepLoc-2.1 (https://services.healthtech.dtu.dk/services/DeepLoc-2.1/ accessed on 10 August 2024); however, there was no direct visual evidence to support the localization of *Sg*SQEs. The ORFs without the termination codon of *Sg*SQEs were fused to the N-terminus of eGFP under the control of the CaMV 35S promoter of the pCambia1300 vector (stored in our laboratory). The 15–25 bp homologous arms were designed using Primer Premier 5.0 for SgSQEs amplification, and the restriction site SalI was chosen for vector digestion. The SgSQE fragments were assembled into the linearized vector using 2x Basic Assembly Mix, and the transformed Trans1-T1 with the recombinant plasmid were selected on LB plates (50 μg/mL kanamycin). The resulting plasmids pCambia1300-*SgSQE*s-*eGFP* were sequenced and then transformed into *Agrobacterium tumefaciens* strain GV3101 chemically competent cells (Tiangen, Beijing, China). The transformants were cultured in LB solid medium (containing 50 μg mL^−1^ kanamycin and 50 μg mL^−1^ rifampicin) at 30 °C for 36 h. Selected colonies were picked and grown in 1 mL liquid medium (200 μL 0.5 M MES buffer, pH 5.6, 4 μL 150 mM acetosyringone), with shaking at 220 rpm for 24 h, and then inoculated into 10 mL of fresh medium at a proportion of 1:100 and similarly grown for 12 h. The cell pellets were collected via centrifugation and resuspended in infiltration buffer (1/2 MS medium, 10 mM MgCl_2_, 10 mM MES, 150 μM acetosyringone) to a final OD_600_ of 1.0. The plasmid expressing HY52-mCherry and the empty vector were used as an ER-marker and negative control, respectively. The suspensions with *Sg*SQEs or the empty vector were mixed with P19 in equal volume and co-infiltrated into 4-to-6-week-old leaves of *N*. *benthamiana*. At 48–72 h after injection, 0.5 cm^2^ infiltrated leaf sections were cut from the agro-infiltrated plants and visualized using a Leica Application Suite X confocal microscope. GFP and mCherry fluorescence signals were captured at wavelengths of 510 nm and 587–610 nm, respectively. Three replications of each experiment were conducted. The primers used for constructing the GFP-fusion recombinant plasmid are listed in Supplementary Information Table S5.

### 5.6. Expression Patterns of SgSQEs in Different Organs and Different Growth Periods of Fruits

Different organs, including roots, stems, leaves, and fruits with different growth periods, were collected in Yongfu County, Guangxi Zhuang Autonomous Region, and frozen with liquid nitrogen immediately. Total RNA was extracted from all samples with Trizol [13] and reverse-transcribed into cDNA using a PrimeScript™ RT Reagent Kit with gDNA Eraser. Real-time PCR was carried out using SYBR Green Supermix (Bio-Rad, Hercules, CA, USA) in a CFX96 real-time system (Bio-Rad), and three biological replicates were performed. The UBQ gene was chosen as an internal control. The calibration curves of the genes all had a single peak, and quantification was calculated using the 2^−ΔΔCt^ method. All experiments were performed in three biological replicates, and the statistical analyses were conducted using GraphPad Prism 8.0.2. All the primers for qRT-PCR are listed in Table 2.

### 5.7. Molecular Docking Analysis and Site-Directed Mutagenesis

AlphaFold2 was employed to conduct homology modeling of *Sg*SQE1 and its mutant with the cofactors FAD and NADPH. The structure of the ligand was retrieved from PubChem (https://pubchem.ncbi.nlm.nih.gov/ accessed on 15 February 2025). The three-dimensional chemical structure of the substrate was mapped using the online software CORINA Classic (https://mn-am.com/products/corina/ accessed on 15 February 2025). All proteins and substrate ligands were stored in pdb format, and the molecules were docked on AutoDock Vina. The docking models and active binding sites were observed using the visual PyMol software 3.1.

The alanine scanning was performed for the candidate amino residues, and the PCR-based site-directed mutagenesis was conducted taking SgSQE1 as the template. The specific primers were designed with mutated bases (Table 1), and all the mutants were introduced into the genome of the strain THY02, followed by an *erg1* knockout using the same method as strain SZ08. The primers used for constructing the SQE1 mutant are listed in Supplementary Information Table S8.

## Figures and Tables

**Table 1:** gRNA for *ERG1* knockout.

gRNA Name	Sequences (5′-3′)
*ERG1*-target gRNA1	GGTGAATTGATGCAACCAGG
*ERG1*-target gRNA2	GGTCAAAGATGGTAATGACA
*ERG1*-target gRNA3	TACTTGAACATGGAAGAACG
*ERG1*-target gRNA4	ATGAGACATCCATTGACTGG
*ERG1*-target gRNA5	TTGGAGAGTTGTAAGCACAA

**Table 2:** Primers for qRT-PCR.

Primers	5′-3′ Sequences
*SgSQE1*-F	GTTCTATCGCATTAGCAGTA
*SgSQE1*-R	GAGGAGCAACAACATTCT
*Sg*SQE2-F	GGTCGCTTATTACTTCCAT
*Sg*SQE2-R	GAACATCTGTCTAACTCCTT

## Data Availability

The original contributions presented in this study are included in the article/Supplementary Materials. Further inquiries can be directed to the corresponding authors.

## Supplementary Materials

The following supporting information can be downloaded at https://www.mdpi.com/article/10.3390/plants14121740/s1.
