# Supplementary material for: Functional Characterization of Squalene Epoxidases from Siraitia grosvenorii
Source: Plants (Basel). 2025 Jun 6;14(12):1740. doi: 10.3390/plants14121740 (PMC12196517; doi:10.3390/plants14121740)
Supplement: Supplementary file 1 [file plants-14-01740-s001.zip › plants-3613094-supplementary.pdf]

Supplementary Information

Supplementary Table S1. All the gene sequences used in this study

Gene Name	5'-3' Sequences
SgSQE1	ATGGTGGATCAGTGCGCGTTAGGCTGGATCTTGGCCTCAGTTCTTGGAGCCGCTGCGC<br>TGTATTTCTTGTTCGGCAGGAAAAACGGCGGAGTTTCGAACGAAAGGAGGCACGAGA<br>GCATAAAGAACATCGCCACCACGAATGGAGAATACAAATCGAGTAACAGCGATGGCG<br>ACATTATCATTGTCGGAGCCGGAGTCGCCGGATCGGCTCTTGCCTATACTCTCGGCAA<br>GGATGGTCGTCGAGTACATGTGATTGAAAGAGACTTGACAGAGCCTGACAGAATTGT<br>TGGTGAATTATTGCAACCTGGGGGCTATCTTAAGTTAACTGAGTTGGGACTTGAAGAT<br>TGTGTGGATGATATTGATGCCCAACGAGTGTATGGTTATGCTCTCTTTAAGGATGGAA<br>AGGATACTAGACTCTCCTACCCCTTGGAAAAATTTCACTCGGATGTGGCTGGGAGGAG<br>CTTTCATAATGGGCGCTTCATTCAGAGGATGCGTGAGAAGGCCGCTAGCCTGCCCAAT<br>GTGAGCTTGGAGCAGGGAACAGTCACTTCTCTGCTCGAGGAAAATGGTATAATTAAA<br>GGTGTGCGGTACAAAACCAAAACTGGCCAAGAGATGACAGCTTATGCACCTCTGACT<br>ATTGTTTGCGATGGGTGCTTTTCAAATTTGCGACGCTCTCTTTGCAACCCTAAGGTAGA<br>TGTTCCATCTTGTTTTGTTGGCCTTGTACTTGAGAATTGCGACCTTCCTTACGCAAATC<br>ACGGACATGTTATACTAGCAGACCCTTCTCCCATTTTGTTCTATCGCATTAGCAGTACA<br>GAGATCCGCTGTCTGGTGGATGTGCCTGGTCAGAAAGTTCCTTCCATTTCAAACGGTG<br>AAATGGCCAACTATTTGAAGAATGTTGTTGCTCCTCAGATTCCTTCTCAACTTTATGAT<br>TCCTTCGTAGCTGCTATTGACAAGGGTAACATCAGAACAATGCCAAATAGAAGTATGC<br>CGGCTGATCCTTATCCTACCCCCGGAGCCCTGTTGATGGGCGATGCATTCAATATGCG<br>CCACCCTCTAACTGGCGGAGGAATGACCGTCGCATTATCTGACGTTGTTGTTCTTAGA<br>GATCTTCTCAAGCCTCTGCGTGATCTGAATGATGCGCCCACTCTCAGCAAGTATCTTG<br>AAGCATTCTACACTCTCCGCAAGCCAGTGGCTTCGACAATAAACACGTTGGCAGGTGC<br>ACTTTACAAGGTCTTTTGTGCCTCACCCGATCAGGCTAGAAAGGAAATGCGTCAGGCT<br>TGCTTCGATTATTTAAGCCTCGGAGGAATCTTCTCGAACGGACCAGTCTCTTTACTCTC<br>TGGCTTAAACCCTCGTCCAATAAGCTTGGTTCTCCATTTCTTTGCGGTGGCGATATACG<br>GTGTTGGCCGGTTGCTGATCCCTTTTCCTTCGCCGAAACGCGTGTGGATCGGAGCGAG<br>AATAATTTCGGGCGCATCGGCCATTATCTTTCCCATTATAAAGGCTGAGGGAGTCAGG<br>CAGATGTTTTTTCCCGCAACTGTTGCTGCTTATTACAGAGCTCCACGTGTCGTGAAAGG<br>GAGGTGA
SgSQE2	ATGGTGGATCAGTGCGCGTTGGGATGGATCTTGGCCTCCGCGCTGGGCCTCGTAATTGC<br>GCTTTGTTTCTTCGTGGCTCCGAGGAGGAATCACAGAGGAGTGGATTCGAAGGAGAGG<br>GACGAGTGCGTCCAAAGCGCTGCAACCACGAAGGGAGAATGCAGATTCAACGATCGC<br>GACGTCGACGTTATCGTCGTTGGCGCCGGTGTTGCCGGTTCCGCTCTTGCTCACACTCT<br>TGGCAAGGATGGTCGTCGAGTTCATGTAATTGAAAGAGACTTGACAGAGCCTGACAGA<br>ATCGTTGGTGAATTATTACAACCTGGGGGTTACCTCAAATTGATTGAATTAGGACTTCAA<br>GACTGCGTCGAGGAGATTGATGCTCAAAGGGTGTATGGCTACGCCCTTTTCAAGGATGG<br>AAAGAACACTCGACTCTCTTACCCATTGGAAAATTTTCACTCTGATGTATCTGGAAGAA<br>GCTTTCACAACGGGCGCTTCATACAGAGAATGAGGGAGAAGGCTGCTTCCCTTCCCAA<br>TGTCAGATTGGAGCAAGGGACAGTTACTTCGCTGCTTGAAGAAAAGGGAACGATCAA<br>AGGTGTGCAGTATAAGTCTAAAAATGGTGAAGAAAAAACAGCATATGCACCTCTGACC<br>ATTGTTTGTGATGGCTGCTTCTCAAACTTGCGCCGCTCTCTCTGCAACCCTATGGTTGAT<br>GTTCCCTCTTATTTTGTGGGATTAGTTCTAGAGAATTGTGAGCTTCCTTTTGCAAATCAC<br>GGGCACGTTATCCTCGGAGATCCTTCTCCCATTTTATTCTACCAGATTAGCAGGACCGAG<br>ATCCGTTGTTTGGTTGATGTTCCTGGTCAGAAGGTTCCTTCTATAGCAAATGGTGAAATG<br>GAGAAATATTTGAAGACTGTAGTAGCTCCTCAGGTTCCCCCGCAAATCTACGATTCCTTT<br>ATCGCTGCTATCGACAAGGGTAATATAAGGACAATGCCAAACAGAAGCATGCCTGCTGC<br>TCCCCACCCAACGCCCGGTGCCTTACTGATGGGTGATGCTTTCAACATGCGCCACCCTC<br>TTACCGGTGGAGGAATGACCGTAGCATTGTCTGATATAGTTGTATTGCGGAACCTCCTCA<br>AGCCTCTGAAGGACTTGAGTGATGCATCTACCCTCTGCAAGTATCTTGAATCCTTTTACA<br>CTTTGCGAAAGCCAGTGGCTTCGACCATCAACACATTGGCAGGGGCATTATACAAGGTC<br>TTTTGTGCATCACCAGATCAAGCTAGGAAGGAAATGCGACAAGCTTGCTTCGATTACTT<br>GAGCCTTGGAGGAATATTCTCAAATGGACCTGTCTCCTTGCTTTCAGGGTTGAATCCTC<br>GCCCCTTAAGTTTGGTTCTCCATTTCTTTGCCGTCGCGATATACGGAGTTGGTCGCTTATT<br>ACTTCCATTTCCTTCAGTGAAAGGCATCTGGATTGGAGCTAGATTGATCTATAGCGCATC<br>AGGTATCATATTCCCAATTATACGGGCGGAAGGAGTTAGACAGATGTTCTTCCCTGCAA<br>CTGTTCCTGCTTATTATAGAAGTCCACCAGTGTTTTGA
SgSQE3	ATGGAATTCCAATCTGAACCATTGTTTGGCGTACTCTTGGCTTCTTTGTTGGCTTTGGTT<br>TTCTTCTTCACTTTGAGAGACGGTACTAAGAACAAGAAGACTACTACTGGTTCTTCTGT<br>TGACTTGAAGAGAACTGACGCTGTTTTGCAAATGTCTCCAGAAAACGACGCTAGAAGA<br>CAAGAAATCATCGGTGACTCTGACGTTATCGTTGTTGGTGCTGGTGTTGCCGGTGCAGC<br>TCTCGCTTACACTCTCGGTAAGGACGGTAGAAAGGTTCACGTTATCGAAAGAGACTTG<br>ACTGAACCAGACAGAATCGTTGGTGAATTGTTGCAACCAGGTGGTTACTTGAAGTTGG

	TTGAATTGGGTTTGGAAGACTCTGTTAAGGGTATCGACGCTCAACAAGTTTTCGGTTAC<br>GCTTTGTACAAGGACGGTAAGCACACTAGATTGACTTACCCATTGGAAAAGTTCGACTC<br>TACTGTTTCTGGTAGATCTTTCCACAACGGTAGATTCATCCAAAGATTGAGAGAATCTGT<br>TAGATTGGAACAAGGTACTGTTACTTCTATCTTGGAAGAAGACGGTACTGTTAAGGGTG<br>TTCAATACAAGACTAAGATCGGTGAAGAATTCACTGCTTACGCTCCATTGACTATCGTTT<br>GTGACGGCGGCTTCTCTAACTTGCGTAGAAACTTGTGTAAGCCACAAATCGACATCCCA<br>TCTTGTTTCGTTGGTTTGGTTTTGGAAAACTGTAAGTTGCCATTCGAAAACCACGGCCA<br>TGTTGTGCTGGCTGACCCATCTCCAATCTTGTTGTACCCAATCTCTTCTACTGAAATCAG<br>ATGTTTGGTTGACATCCCAGGTCAAAAGGTTCCATCTGTTGCTAACGGTGAAATGGCTA<br>GATACTTGAAGACTGTTGTTGCTCCACAAGTTCCACCAGAATTGCACGCTGCTTTCATC<br>GCTGCTATCGAAAAGGGTAACATCAAGTCTACTACTAACAGATCTATGCCAGCTGCTCC<br>ACACCCAACTCCAGGTGCTTTGTTGCTCGGCGACGCGTTCAACATGAGACATCCATTGA<br>CTGGTGGTGGTATGACTGTTGCTTTGGCTGACATCGTTGTTTTGAGAGACTTGTTGAGA<br>CCATTGGCTAACTTGAAGGACGCTGACGCTTTGTGTCACTACTTGGAATCTTTCTACAC<br>TTTGAGAAAGCCAGTTGCTTCTACTATCAACACTTTGGCTGGTGCGCTCTACAAGGTGT<br>TCTGCGCTTCTCCAGACTCTGCTAGAAAGGAAATGAGAGAAGCTTGTTTCGACTACTTG<br>TCTTTGGGTGGTGTTTTCTCTTCTGGTCCAGTTGCTTTGTTGTCTGGTTTGAACCCAAGA<br>CCATTGTCTTTGTTCTGTCACTTCTTCGCTGTTGCTATCTACGGTGTTTCTAGATTGTTGA<br>TCCCATTCCCATCTCCAATGAGAATCTGGATCGGTGTTAGATTGATCACTGTTGCTGCTG<br>GTATCATCTTCCCAATCATCAAGGCTGAAGGTGTTAGACAAATGTTCTTCCCAGCTACTG<br>TTCCAGCTTACTACAGAGCTCCACCAATGTGA
SgSQE4	ATGTTGCAAATCTTCTCTGGTCACTCTTCTTGGCAAATCTCTTTCGAATTCTCTCACCAC<br>TTCTTGTCTCCAATGGAAAACTTGCACCACTTTGGCGGCTTCTTGAACTGGAAGAAGA<br>AGAGAAAGAAGTCTCAAACTAGAGCTTTCGTTGAAGTTAGAGCTTCTGGTTGTGTTAA<br>GGCTTCTCCAGAAAACTGTGTTTGTAGACCAGCTGTTGAAGGTAACAGAGACATCGTT<br>GTTGTTGGTGCTGGTGTTGCCGGTGCAGCTCTCGCTTACACTCTCAGAAAGGACGGTA<br>GAAAGGTTCACGTTATCGAAAGAGACTTGGAACCAGAAAGAATCGTTGGTGAATTGTT<br>GCAACCAGAATTGTTGCAACCAGGTGGTTACTTGAAGTTGATCGAATTGGGTATCGAAG<br>ACTGTGTTAGAGAAATCGACGCTCAAAAGGTTGGTAAGAACACTAACTTGGCTTACCC<br>ATTGCAAAACTTCGACTCGGAAGTAAGCGGTAGATCTTTCCACAACGGTAGATTCATCC<br>AAAGATTGAGAGAAAAGGCTGCTTCTTTGCCAAAGGTTAAGTTGGAACAAGGTACTGT<br>TACTTCTTTGTTGGAAGAAAACGGTATCGTTAAGGGTGTTCAATACAAGACTAAGGTTG<br>GTCAAGACTTGAAGGCTTACGCTCCATTGACTATCGTATGTGACGGTTGCTTCAGCAAC<br>TTGAGACACTCTTTGTGTAACCCAAAGGTTGAAATCCCATCTTGTTTCGTTGGTATCATC<br>TTGGAAAACTGTAACTTGCCATACACTAACCACGGTCACGTTATCTTGGAAAACCCATC<br>TCCAATCTTGTTCTACCCAATCTCTTCTACTGAAATCCAATGTTTGGTTGACGTTCCAGG<br>TCAAAAGGTTCCATCTGTTGCTAACGGTGAAATGGCTGACTACTTGAGAAACGTTGTTG<br>CTCCACAAGTTCCACCACAATTGCACAACTCTTTCATCGCTGCTATCGAAAAGGGTAAG<br>ATCAAGTCTATGACTAACAGATCTATGCCAGCTGCTCCACACTCTACTGCTGGTGCTTTG<br>TTGATGGGTGACGCTTTCAACATGAGACACCCATTGACTAGAGGTGGTATGACTGTTGC<br>TTTGTCTGACATCGTTATCTTGAGAGACTTGCACAGACCATTGCAAGACTTGGACGACG<br>CTGCTGCTATCTGTAAGTACTTGGAATCTTTCTACACTTTGAGAAAGCCAGTTGCTTCTA<br>CTATCAACACTTTGGCTGGTGCGCTCTACACTGTGTTCTGCGCTTCTCCAGACCCAGCT<br>AGAACTGAAATGAGACACGCTTGTTTCGACTACTTGTCTTTGGGTGGTGCTTTCTCTTC<br>TAGACCAATGTCTTTGTTGTCTGGTTTGCACCCAAGACCACTCTCGTTGGTATTCCACTT<br>CTTCGCTGTTGCTGTTTACGGTGTTGGTAGATTGTTGTTGCCATTCCCATCTCCAAAGTC<br>TTTGTGGTTGGGTGCTAGATTGATCTACGTTGCTTCTTCTATCATCTTCCCAATCATCAAG<br>GCTGAAGGTGTTAGACAAATGTTCTTCCCAGTTACTGTTCCAGCTTACTACAGAGCTCC<br>ACCAATCTGA
SgSQE5	ATGGAAAACTTGCACCACTTTGGCGGCTTCTTGAACTGGAAGAAGAAGAGAAAGAAG<br>TCTCAAACCAGAGCTTTCGTCGAAGTTAGAGCTTCTGGTTGTGTTAAGGCCTCTCCAGA<br>AAACTGTGTCTGTAGACCAGCTGTTGAAGGTAACAGAGACATTGTCGTCGTCGGTGCC<br>GGTGTTGCGGGGGCTGCTCTCGCTTACACTCTCAGAAAGTTCACCATGAACGAAAAGT<br>TGCAAAAGTACTTGTCTTTGAACAAGTCTCACGTTGTTGCTGCTGATTGTGTCAGAGAA<br>ATTGATGCTCAAAAGGTTGAAAGAACTTTGACTTGGTTGATCCCATGTAAGATTTTGAT<br>TCAAAAGTTCCAAGAAGAAGCTTTCATAATGGCTGTATGCGATGGTTGTTTCTCTAACT<br>TGAGACACTCTTTGTGTAACCCAAAGGTTGAAATTCCATCTTGTTTCGTTGGTATCATCT<br>TGGAAAACTGTAACTTGCCATACACTAACCACGGTCACGTTATCTTGGAAAACCCATCT<br>CCAATCTTGTTCTACCCAATCTCTTCTACCGAAATCCAATGTTTGGTCGATGTCCCAGGT<br>CAAAAGGTTCCATCTGTTGCCAACGGTGAAATGGCTGACTACTTGAGAAACGTTGTTG<br>CCCCACAAGTTCCACCACAATTGCACAACTCCTTCATTGCTGCTATTGAAAAGGGTAAG<br>ATCAAGTCTATGACTAACAGATCTATGCCAGCTGCTCCACACTCTACTGCTGGTGCCTTG<br>TTGATGGGTGATGCTTTCAACATGAGACACCCATTGACCAGAGGTGGTATGACTGTTGC<br>TTTGTCTGACATTGTCATCTTGAGAGACTTGCACAGACCACTCCAAGATCTCGATGATG<br>CTGCGGCTATCTGTAAGTACTTGGAATCCTTCTACACCTTGAGAAAGCCAGTTGCTTCC<br>ACTATCAACACTTTGGCTGGTGCCTTGTACACTGTCTTCTGTGCTTCTCCAGATCCAGCT<br>AGAACTGAAATGAGACACGCTTGTTTCGACTACTTGTCTTTGGGTGGTGCTTTCTCTTC

	TAGACCAATGTCTTTGTTGTCCGGTTTGCACCCAAGACCACTCTCGTTGGTATTCCACTT CTTCGCTGTTGCTGTTTACGGTGTTGGTAGATTGTTGTTGCCATTCCCATCCCCAAAGTC TTTGTGGTTGGGTGCTAGATTGATCTACGTTGCTTCTTCTATTATTTTCCCAATCATTAAG GCTGAAGGTGTTAGACAAATGTTCTTCCCAGTCACTGTTCCAGCTTACTACAGAGCTCC ACCAATCAAGAAGATGGAAAACCAAAAGGATCAAAAGAGAACCTGTTGA
SgSQE6	ATGGCTATTACTATGGATACTTTGGCCGCTTTGGTTCAATTGGCTTTCTGTAACCACTGG TCTGGTTTGATGAGAGAAGCTAACGTTAAGGAAACTTCTTTCAGATTCTTGTGTTTGGG TGGTGTTTGTGTTAACGCCTTGATGTCTTTGATCTCTGGTTTGAACCCAAACACTTCCAC TTTGTTGTTGTTGTTGTTGGCCATGGCTGTCTACAACTCTATTGCTTCTTCCATTTCCTTC TCTTGA
SgSQE7	ATGATTTACCAATTCGTTGTCTTGGGTTTGATCGCCTCTCCATTGGCTTTGTTCGTTTTGA AGAAGCAATCTCAAAGAAAGCAATCTCAATCTTTCTTGCAAAACCAACAACCAAAGTC TCCAGCTAACGGTTCTTCCATTTGTAGATCTAAGTCTCACGGTAGAATTGATGTTGTCAT TGTTGGTGCCGGCGTTACTGGCTCTGCTCTCGCTTACGCTCTCGGTAAGGATGGTAGAA GAACTCACATCATTGAACAAGATTTGACCATGCACAACAGAATGATCTGGGAAGTTTTG AAGCCAGGTGGTTACTTGAAGTTGTTCGAATTGGGTTTGGAAGATTGTGTTTTGGATAT TGAAGCTCAACAAGTTTTCGGTTACGTCATCTACAAGGATGGTAAGTACATTAGATTGC CATACAGATTGAACTCTTCCAACTCTAACGTTACTGAAAGATCTTTCCACAACGGTTTG TTCGTTCAAAGATTGAGAGAAAAGGCTGCTTCTTTGAGAAACGTTGAATTCAAGCAAG GGACTGTCTTGGGCTTGTTGAAGCAAAAGGATACTGTCAAGGGTGTTAGATACAAGAC TAAGACCGGTCAAGAAATGGTTGCTCACGCTGATTTGACTGTCATCTGTGACGGTAGAT ACTCTAACTTGAGAAGATTCTTGTGTAGACACAAGGTTAGATTGGTTACTCCAAAGATC AGATGTTTCGTTGACATCCCAGGTCACAAGGCTCCATCCATTTCCAACGGTGAAATGCC ACACTACTTGAAGACTATGGTTGCTCCACAAATCCCACCAGAATTCTACGATGCTTTCAT GTCTACTATCGATAAGGCTACCATGAGAATCATCCCATACTGGAAGATGCCACCACACC CAAACCCAACCGCCGGTGCCATTTTCTTGGGTGACGCTTGA
P_{TEF1}	ATAGCTTCAAAATGTTTCTACTCCTTTTTTACTCTTCCAGATTTTCTCGGACTCCGCGCAT CGCCGTACCACTTCAAAACACCCAAGCACAGCATACTAAATTTCCCCTCTTTCTTCCTCT AGGGTGTCGTTAATTACCCGTACTAAAGGTTTGGAAAAGAAAAAAGAGACCGCCTCGT TTCTTTTTCTTCGTCGAAAAAGGCAATAAAAATTTTTATCACGTTTCTTTTTCTTGAAAA TTTTTTTTTTTGATTTTTTTCTCTTTCGATGACCTCCCATTGATATTTAAGTTAATAAACGG TCTTCAATTTCTCAAGTTTCAGTTTCATTTTTCTTGTTCTATTACAACTTTTTTTACTTCTT GCTCATTAGAAAGAAAGCATAGCAATCTAATCTAAGTTTTAATTACAAA
T_{CYC1}	ATCCGCTCTAACCGAAAAGGAAGGAGTTAGACAACCTGAAGTCTAGGTCCCTATTTATT TTTTTATAGTTATGTTAGTATTAAGAACGTTATTTATATTTCAAATTTTTCTTTTTTTTCTGT ACAGACGCGTGTACGCATGTAACATTATACTGAAAACCTTGCTTGAGAAGGTTTTGGGA CGCTCGAAG
XI-3 up	AGTTACTTGCTCTATGCGTTTGCGCATCCTCTTTTTACTTTTTTTTTTTCAGTAAAGCCTA AGCATAAATCGTTTTATACGTACGACACGTTCAACTTTTCTTGGTTAGTAGTGGCAATCT CTGCAATACATACAGGGAGTCATGGTCTATCATCTTGTCCAATCAAAGAAGCATCGGTT CAGATCGAGCAAACTGTAGGGAGAAAGGAAAGTAGAAATGCAGAGTGTGCTATATGTC CAATCTCGGTTTTGTAGTTTGGATGTCATTAGAGATCTACCACCCAACCGGCTGCTTTCA TGTGGAACAGAAAAGAAATCGGGGCGCTTCCTCTTCTGTATTCCTTTAATTAACGTTTTT ATTCAGCCATCTAACCATCATACCCCCATACGGTAACAAAACCTCTTCTAAGAAAAGAA GTCTCTGCTCCTCCGCCATCTTATTTTTATTCGCTGCGCGCGTTTATTGTCGCATCGCTAG CCAGCAAAAAGTTGGTTGCCTTTTTTTACCTAAAAAAGACACATCTAACTGATTAGTTT TCCGTTTTAGGATATTGACGCCAAGCGTGCGTCTGATTTCTACG
XI-3 down	GGGCCATTTTATTTTTGAGGATTGGGCTGATCATTTTCCTTACGTGGATTGAGCCAGCAA TACAGATCATTATTAAACTGTTTTGTACATGATGTTAGTATATAATCGTAAAGCTTTTCTAA TATGTATACCTTATACATGGAACTCCACAGAACTTGCAAACATACCAAAAATCCTTTATT CTTGTTCACTCATTTTACATCAAAAAATAATATTTCAGTTATTAAGGAAAATAAAAAAAT AGATTAGAGAAGCATTTTGAAGAAATAGTATATTCTTTTATTGAACCTAAGAGCGTGATA TTTTTACTCGAAATAAAATACGAAAAATCTATACACTCATCTTTCCGACTACTATTGGCTC CTGCTCAAAAAAAGAGGGAAAAAAAGCTCCAAAATTCTATCTTTTCCTATCGCTCCTGT CCTATCCTTATTACGTTCATTACTATTTTAATACTATCCATTCTTTTATTTTCAGTCTAAAA AAAACATTTCTCATAACGGGAAAAGCAAAAAAATGTCAAGCTTATACATCAAAACACC ACTGCATGCATTATCTGCTGGTCCGGATTCTCATG
Δ*erg1 up*	TACTGCCGTAGCGGGCCTTCGTATAGCTCGGCCGAGCTCGTACAAAAGGCAAGCAGTG TATCGGACAGAGCTGATATAACACAATACGCTCGTAGTCGATGCATGCCGTGGCTGCTC TCGGTCGGGTATAAGTCTTAGACAATAGTCTTACCTCGCATGTATAATAAATCTTTTGTAT TTAATCTATTATATGTTTCTATGCTTTTTTTTCCTATTGTTGTTTGCTTTTCCTTTTCCTTAT TTCTTTCTAGCTTCTAATTTCTTTCTTTTTTTTTTTTTTTTTCATTGAAAATTATATATATATA TATATATCAGAACAATTGTCCAGTATTGAACAATACAGGTTATTTCGAACAATTGAAAAA AAAAAATCACAGAAAAACATATCGAGAAAAGGGTC
Δ*erg1 down*	CTACAGCTTATAAGGGAGAGAGGATAGGAACCGTCAAACATTAAGCTGCACCTTTTTTT TTTATTACAGAAGTCGGCTTGGAAGGCTTGTATAGTACATTACGATAAACACATCTTATT

	TTTATTTATTACTTATTTATTTTACATATTTTCAAAAAAATTCACATATCATTTATTATTAAC<br>CGAAGTGTTTTATACTTTTTGTTCTTTCCTTAAAAATGCCTCCAACAGAAAAGGAAAAA<br>TGGCTTGATATGCTTCAAAATATGCATTGAGATTCATATTCGGTTATCAATTAACGATATT<br>ACTTCCTTAAGTGATATTAAATCAAGCTTGCCGGATTCTGCAGTCAGAATTATACATAAT<br>AGTATTTGTGCTCTCGTAAATCCTTTCAGAATTACACC
pHXT1	GCCACAATGAAACTTCAATTCATATCGACCGACTATTTTTCTCCGAACCAAAAAAATAG<br>CAGGGCGAGATTGGAGCTGCGGAAAAAAGAGGAAAAAATTTTTTCGTAGTTTTCTTGT<br>GCAAATTAGGGTGTAAGGTTTCTAGGGCTTATTGGTTCAAGCAGAAGAGACAACAATT<br>GTAGGTCCTAAATTCAAGGCGGATGTAAGGAGTATTGGTTTCGAAAGTTTTTCCGAAGC<br>GGCATGGCAGGGACTACTTGCGCATGCGCTCGGATTATCTTCATTTTTGCTTGCAAAAA<br>CGTAGAATCATGGTAAATTACATGAAGAATTCTCTTTTTTTTTTTTTTTTTTTTTTTTTTAC<br>CTCTAAAGAGTGTTGACCAACTGAAAAAACCCTTCTTCAAGAGAGTTAAACTAAGACT<br>AACCATCATAACTTCCAAGGAATTAATCGATATCTTGCACTCCTGATTTTTCTTCAAAGA<br>GACAGCGCAAAGGATTATGACACTGTTGCATTGAGTCAAAAGTTTTTCCGAAGTGACC<br>CAGTGCTCTTTTTTTTTTTCCGTGAAGGACTGACAAATATGCGCACAAGATCCAATACGT<br>AATGGAAATTCGGAAAAACTAGGAAGAAATGCTGCAGGGCATTGCCGTGCCGATCTTT<br>TGTCTTTCAGATATATGAGAAAAAGAATATTCATCAAGTGCTGATAGAAGAATACCACTC<br>ATATGACGTGGGCAGAAGACAGCAAACGTAAACATGAGCTGCTGCGACATTTGATGGC<br>TTTTATCCGACAAGCCAGGAAACTCCACCATTATCTAATGTAGCAAAATATTTCTTAACA<br>CCCGAAGTTGCGTGTCCCCCTCACGTTTTTAATCATTTGAATTAGTATATTGAAATTATAT<br>ATAAAGGCAACAATGTCCCCATAATCAATTCCATCTGGGGTCTCATGTTCTTTCCCCACC<br>TTAAAATCTATAAAGATATCATAATCGTCAACTAGTTGATATACGTAAAATC

Supplementary Table S2. Primers used for integration of exogenous *SQE* at XI-3 site.

primers	5'-3' Sequences
XI-3-up-F	AGTTACTTGCTCTATGCGTTTGCGC
XI-3-up-R	AGGAGTAGAAACATTTTGAAGCTATCGTAGAAATCAGACGCACGCTTGGC
XI-3-TEF1-F1	GCCAAGCGTGCGTCTGATTTCTACGATAGCTTCAAAATGTTTCTACTCCT
XI-3-TEF1-R1	TAACGCGCACTGATCCACCATCGGGTTTGTAATTAAAACTTAGATTAGATTGC
XI-3-SQE1-F1	GCAATCTAATCTAAGTTTTAATTACAAACCCGATGGTGGATCAGTGCGCGTTA
XI-3-SQE1-R1	CCAATCCTCAAAAATAAAATGGCCCCTTCGAGCGTCCCAAAACCTTCTC
XI-3-TEF1-F2	GCCAAGCGTGCGTCTGATTTCTACGATAGCTTCAAAATGTTTCTACTCCT
XI-3-TEF1-R2	AACGCGCACTGATCCACCATCGGGTTTGTAATTAAAACTTAGATTAGATT
XI-3-SQE2-F2	AATCTAATCTAAGTTTTAATTACAAACCCGATGGTGGATCAGTGCGCGTT
XI-3-SQE2-R2	CCAATCCTCAAAAATAAAATGGCCCCTTCGAGCGTCCCAAAACCTTCTC
XI-3-TEF1-F3	GCCAAGCGTGCGTCTGATTTCTACGATAGCTTCAAAATGTTTCTACTCCT
XI-3-TEF1-R3	CCAAACAATGGTTCAGATTGGAATTCCATCGGGTTTGTAATTAAAACTTAGATTA<br>GATTGC
XI-3-SQE3-F3	GCAATCTAATCTAAGTTTTAATTACAAACCCGATGGAATTCCAATCTGAACCATT<br>GTTTGG
XI-3-SQE3-R3	CCAATCCTCAAAAATAAAATGGCCCCTTCGAGCGTCCCAAAACCTTCTC
XI-3-TEF1-F4	GCCAAGCGTGCGTCTGATTTCTACGATAGCTTCAAAATGTTTCTACTCCT
XI-3-TEF1-R4	GTGACCAGAGAAGATTTGCAACATCGGGTTTGTAATTAAAACTTAGATTAGATT<br>GC

XI-3-SQE4-F4	GCAATCTAATCTAAGTTTTAATTACAAACCCGATGTTGCAAATCTTCTCTGGTCAC
XI-3-SQE4-R4	CCAATCCTCAAAAATAAAATGGCCCCTTCGAGCGTCCCAAAACCTTCTC
XI-3-TEF1-F5	GCCAAGCGTGCGTCTGATTTCTACGATAGCTTCAAAATGTTTCTACTCCT
XI-3-TEF1-R5	CCAAAGTGGTGCAAGTTTTCCATCGGGTTTGTAATTAAAACTTAGATTAGATTGC
XI-3-SQE5-F5	GCAATCTAATCTAAGTTTTAATTACAAACCCGATGGAAAACTTGCACCACTTTGG
XI-3-SQE5-R5	CCAATCCTCAAAAATAAAATGGCCCCTTCGAGCGTCCCAAAACCTTCTC
XI-3-TEF1-F6	GCCAAGCGTGCGTCTGATTTCTACGATAGCTTCAAAATGTTTCTACTCCT
XI-3-TEF1-R6	CCAAAGTATCCATAGTAATAGCCATCGGGTTTGTAATTAAAACTTAGATTAGATTGC
XI-3-SQE6-F6	GCAATCTAATCTAAGTTTTAATTACAAACCCGATGGCTATTACTATGGATACTTTGG
XI-3-SQE6-R6	CCAATCCTCAAAAATAAAATGGCCCCTTCGAGCGTCCCAAAACCTTCTC
XI-3-TEF1-F7	GCCAAGCGTGCGTCTGATTTCTACGATAGCTTCAAAATGTTTCTACTCCT
XI-3-TEF1-R7	CCAAGACAACGAATTGGTAAATCATCGGGTTTGTAATTAAAACTTAGATTAGATTGC
XI-3-SQE7-F7	GCAATCTAATCTAAGTTTTAATTACAAACCCGATGATTTACCAATTCGTTGTCTTGG
XI-3-SQE7-R7	CCAATCCTCAAAAATAAAATGGCCCCTTCGAGCGTCCCAAAACCTTCTC
XI-3-OsSQE1-F2	AATCTAAGTTTTAATTACAAACCCGATGGTTGACCCCTATGCCGTCGGAT
XI-3-OsSQE1-R2	TTCGGAAATCAACTTCTGTTCCATGTTAAGCAGCTGGTGGAGTACGGTAG
XI-3-Cd-F3	CTACCGTACTCCACCAGCTGCTTAACATGGAACAGAAGTTGATTTCCG
XI-3-Cd-R3	CATGAGAATCCGGACCAGCAGAT
XI-3-colony-F1	CGTTTTGCCTGACGATCAGCCTGGT
XI-3-colony-R1	CTTTTCCAAACCTTTAGTACGGGTA
XI-3-colony-F2	AGAAGTTGATTTCCGAAGAAGACCT
XI-3-colony-R2	TTGAGCGAATGAAACGAATACTTCC

Supplementary Table S3. Primers used for *ERG1* gene knockout.

primers	5'-3' Sequences
ERG1-up-F	TACTGCCGTAGCGGGCCTTCGTATA
ERG1-up-R	ATCCTCTCTCCCTTATAAGCTGTAGGACCCTTTTCTCGATATGTTTTTCT
ERG1-down-F	AGAAAAACATATCGAGAAAAGGGTCCTACAGCTTATAAGGGAGAGAGGAT
ERG1-down-R	GGTGTAATTCTGAAAGGATTTACGA
ERG1-colony-F	CTTGATTGGTCTGGGGGT
ERG1-colony-R	TTCCAATAATAGCGCCACC

Supplementary Table S4. Primers used for replacement the *pERG7* with pHXT1.

primers	5'-3' Sequences
pHXT1-F	AAGGTGAAAGAATCACATATATAACGGCAAATTACTGGGATTGCTGTTGTGCC<br>ACAATGAAACTTCAATTCATATCG
pHXT1-R	CGTGGATCTGTCTTTGGTAGACCGATTGTGTCAGAATAAAATTCTGTCATGATT<br>TTACGTATATCAACTAGTTGACGATT
pHXT1-colony-F1	ACGACAGTATGTAAAGATAAAAGGGAG
pHXT1-colony-R1	CATATTTGTCAGTCCTTCACGG
pHXT1-colony-F2	CAAGGCGGATGTAAGGAGTAT
pHXT1-colony-R2	GGCGATATAGTTTACGGCT

Supplementary Table S5. Primers used for constructing GFP-fusion recombinant plasmid.

primers	5'-3' Sequences
SgSQE1-GFP SalI-F	GAAAGCTTCTGCAGGGGCCCGGGGTCGACATGGTGGATCAGTGCGCG
SgSQE1-GFP SalI-R	ACCGGATCCACTAGTATTTAAATGTCGACCCCTCCCTTTCACGACACG
SgSQE2-GFP SalI-F	GAAAGCTTCTGCAGGGGCCCGGGGTCGACATGGTGGATCAGTGCGCG
SgSQE2-GFP SalI-R	ACCGGATCCACTAGTATTTAAATGTCGACCAAACACTGGTGGACTTCT
SgSQE3-GFP SalI-F	GAAAGCTTCTGCAGGGGCCCGGGGTCGACATGGAATTCCAATCTGAAC
SgSQE3-GFP SalI-R	ACCGGATCCACTAGTATTTAAATGTCGACCCATTGGTGGAGCTCTG
SgSQE4-GFP SalI-F	GAAAGCTTCTGCAGGGGCCCGGGGTCGACATGTTGCAAATCTTCTCTG
SgSQE4-GFP SalI-R	ACCGGATCCACTAGTATTTAAATGTCGACCGATTGGTGGAGCTCTGTAG
SgSQE5-GFP SalI-F	GAAAGCTTCTGCAGGGGCCCGGGGTCGACATGGAAAACTTGCACCAC
SgSQE5-GFP SalI-R	ACCGGATCCACTAGTATTTAAATGTCGACCACAGGTTCTCTTTTGATC
SgSQE6-GFP SalI-F	GAAAGCTTCTGCAGGGGCCCGGGGTCGACATGGCTATTACTATGGATAC
SgSQE6-GFP SalI-R	ACCGGATCCACTAGTATTTAAATGTCGACCAGAGAAGGAAATGGAAGAAG
SgSQE7-GFP SalI-F	GAAAGCTTCTGCAGGGGCCCGGGGTCGACATGATTTATCAGTTTGTG
SgSQE7-GFP SalI-R	ACCGGATCCACTAGTATTTAAATGTCGACCAGCGTCACCCAAGAAAAT

Note: The underscores in the table represent the homologous arms in the primers.

Supplementary Table S6. Primers used for constructing SQE over-expression vector.

primers	5'-3' Sequences
plas-Gal1-F	CCGCGGCTAGCTAAGATCCGCTCTAACCGA
plas-Gal1-R	GTCGACGCCCGGGCCCTATAGTGAGTCGTA
SgSQE1-Gal1-F	TAGGGCCCGGGCGTCGACATGGTGGATCAGTGCGCGTTAGGC
SgSQE1-Gal1-R	GATCTTAGCTAGCCGCGGTCACCTCCCTTTCACGACACGTGG
SgSQE2-Gal1-F	TAGGGCCCGGGCGTCGACATGGTGGATCAGTGCGCGTTGGGA
SgSQE2-Gal1-R	GATCTTAGCTAGCCGCGGTCAAAACACTGGTGGACTTCTATA
plas-Gal1-F’	GCTAGCTAAGATCCGCTCTAACCGAAAAGG
plas-Gal1-R’	GTCGACGCCCGGGCCCTATAGTGAGTCGTA
ERG1-Gal1-F	TCACTATAGGGCCCGGGCGTCGACATGTCTGCTGTTAACGTTGCACCT

ERG1-Gal1-R	CGGTTAGAGCGGATCTTAGCTAGCTTAACCAATCAACTCACCAAAC
OsSQE1-Gal1-F	TCACTATAGGGCCCGGGCGTCGACATGGTTGACCCCTATGCCGTCGG
OsSQE1-Gal1-R	CGGTTAGAGCGGATCTTAGCTAGCTTAAGCAGCTGGTGGAGTACGGT
GAL1-colony-F	TTTAACGTCAAGGAGAAAAAACCCC
GAL1-colony-R	CTAACTCCTTCCTTTCGGTTAGAGC

Supplementary Table S7. Primers used for constructing the OsONS1-SQE over-expression vector.

**primers**	**5'-3' Sequences**
plas-Gal10-F1	GCTAGAGTAAGTAGTTCGCCAGTTA
plas-Gal10-R1	CCATTGTTCCACCAAGAATGAGAGCTCTTAATTAACAATTCT
plas-Gal10-F2	AGCAGTTTTCAACTTCCACATGCGGCCGCCCTTTAGTGAGGG
plas-Gal10-R2	ACTATTAACTGGCGAACTACTTACT
OsONS1-Gal10-F3	AGAATTGTTAATTAAGAGCTCTCATTCTTGGTGGAACAATGG
OsONS1-Gal10-R3	CCCTCACTAAAGGGCGGCCGCATGTGGAAGTTGAAAACTGCT

Supplementary Table S8. Primers used for constructing *Sg*SQE1 mutant.

**primers**	**5'-3' Sequences**
I95A-F	ACAGAGCCTGACAGAGCTGTTGGTGAAT
I95A-R	GCTCTGTCAGGCTCTGTCAAGTCTCT
E98A-F	GACAGAATTGTTGGTGCATTATTGCAAC
E98A-R	GCACCAACAATTCTGTCAGGCTCTG
L99A-F	AGAATTGTTGGTGAAGCATTGCAACCT
L99A-R	GCTTCACCAACAATTCTGTCAGGCTC
Y265A-F	TCTCCCATTTTGTTCGCTCGCATTAGC
Y265A-R	GCGAACAAAATGGGAGAAGGGTCTGC
L275A-F	ACAGAGATCCGCTGTGCGGTGGATGTG
L275A-R	GCACAGCGGATCTCTGTACTGCTAAT
M323A-F	GGTAACATCAGAACAGCGCCAAATAGAAG
M323A-R	GCTGTTCTGATGTTACCCTTGTCAAT
P350A-F	TTCAATATGCGCCACGCTCTAACTGG
P350A-R	CGTGGCGCATATTGAATGCATCGCC
G353A-F	GCCACCCTCTAACTGCCGGAGGAAT
G353A-R	GCAGTTAGAGGGTGGCGCATATTGAAT

Note: The underscores in the table indicate the positions mutated to alanine.
